# Direct Determination of Flavanone Isomers in Citrus Juice by Paper Spray Tandem Mass Spectrometry

**DOI:** 10.3390/antiox14010020

**Published:** 2024-12-27

**Authors:** Lucia Bartella, Fabio Mazzotti, Ilaria Santoro, Ines Rosita Talarico, Leonardo Di Donna

**Affiliations:** 1 Dipartimento di Chimica e Tecnologie Chimiche, Università Della Calabria, Via Pietro Bucci Cubo 12/D, I-87030 Rende, CS, Italy; lucia.bartella@unical.it (L.B.); fmazzotti@unical.it (F.M.); inesrosita.talarico@unical.it (I.R.T.); 2 QUASIORA Laboratory, AGRINFRA Research Net, Università Della Calabria, Via Pietro Bucci Cubo 12/D, I-87036 Rende, CS, Italy; ilaria.santoro@unical.it

**Keywords:** flavanone isomers, antioxidant compounds, paper spray ionization, tandem mass spectrometry, citrus juices

## Abstract

A novel and efficient analytical protocol based on paper spray tandem mass spectrometry was developed for the determination of isomeric *O*-glycoside flavanones in citrus juices and beverages. This approach significantly reduces sample preparation time and solvent consumption compared to traditional chromatographic techniques. By exploiting the unique fragmentation patterns of these compounds, accurate quantification of both diglycosides and their individual isomers (neohesperidoside and rutinose derivatives) was achieved. The method demonstrated excellent analytical performance, with high accuracy, selectivity, and reproducibility. The impact of matrix effects was mitigated through the construction of ratio calibration curves, ensuring reliable quantification in complex matrices. Finally, a simple DPPH experiment to assay the antioxidant activity of each single positional isomer was performed, indicating the superior ability of neohesperidose conjugates. This simplified method offers a valuable tool for quality control, authenticity assessment and the study of health benefits associated with citrus consumption.

## 1. Introduction

Flavanones, a distinct subclass within the flavonoid family, are naturally present in high quantities in citrus fruits, tomatoes, and some aromatic herbs [1,2]. These bioactive polyphenolic compounds have attracted considerable scientific attention due to their wide array of potential health advantages and therapeutic attributes [3]. They are structurally defined by a 2,3-dihydro-2-phenylchromen-4-one backbone and include notable compounds such as naringenin, hesperetin, and eriodictyol often linked to a diglycoside moiety. Recent research highlights flavanones’ impressive pharmacological profile, underlying their antioxidant, anti-inflammatory, antimicrobial, and anticancer activities [3,4]. Their potent antioxidant properties help in neutralizing harmful free radicals, thereby protecting cells from oxidative stress and reducing the risk of chronic diseases [5,6,7].

Flavanones’ anti-inflammatory effects are particularly promising in the management of inflammatory conditions, potentially offering natural alternatives to synthetic anti-inflammatory drugs. Moreover, flavanones exhibit significant antimicrobial properties, making them effective against a broad spectrum of pathogens, including bacteria, viruses, and fungi. This antimicrobial activity is especially relevant in the context of rising antibiotic resistance, presenting flavanones as potential candidates for new antimicrobial therapies [8]. Their potential anticancer properties are also reported, as flavanones have been shown to inhibit the proliferation of cancer cells, induce apoptosis, and interfere with various signaling pathways involved in tumor growth and metastasis [9]. In addition to these therapeutic benefits, flavanones also contribute to cardiovascular health by improving blood vessel function, reducing blood pressure, and preventing atherosclerosis. Their ability to modulate lipid metabolism and insulin sensitivity further underscores their potential role in managing metabolic disorders such as obesity and diabetes [10].

Overall, flavanones represent a promising class of bioactive compounds with multifaceted health benefits. Continued research into their mechanisms of action and therapeutic applications holds great promise for the development of novel nutraceuticals and pharmaceuticals aimed at improving human health and combating various diseases [11]. The analytical methods largely used for the quantification of flavanones are based on HPLC separation coupled with different detectors. Among the most widely used is the UV detector, as the aglycone moiety of these molecules responds quite well in the near UV wavelength range [12], and mass spectrometry, which may give more specificity to the analysis, especially if it relies on the detection of fragment ions [13,14,15,16,17,18,19,20]. Even if HPLC has the undoubted advantage of separating analytes by their retention times, the analyses are unavoidably quite time consuming, especially when optimum chromatographic resolution is required, such in the case of isomers separation. Paper spray mass spectrometry (PS-MS) is generally used in food analysis to provide information on molecular profiles of food components [21,22,23,24,25]; like any other ambient mass spectrometry technique, it possesses the advantage of significantly reducing the time of analysis. It has also been successfully applied to the quantification of quality markers of foods and to test food safety [26,27,28,29]. Here, we present an innovative and very rapid analytical method based on paper spray tandem mass spectrometry, which quantifies diglycosides flavanones, giving, at the same time, information on the relative amounts of the single isomers. The isomer profile and its relative amounts can be used to characterize citrus juices, allowing for the distinction between different types of citrus fruit and detect any adulteration, such as the addition of undeclared juices. This information is crucial for ensuring the authenticity and quality of the product. In addition, the antioxidant activity of the flavanones was examined to determine any differences between the isomer pairs, which could imply varying nutraceutical properties.

## 2. Materials and Methods

### 2.1. Chemicals

All solvents used were HPLC grade and commercially available (Sigma-Aldrich, St. Louis, MO, USA). Pure flavanone-*O*-glycoside standards (both neohesperidosides and rutinosides) were purchased from Extrasynthese (Genay Cedex, France).

### 2.2. Real Samples

The developed method was applied to seven citrus drinks, including four freshly squeezed juices (citron, orange, grapefruit, and bergamot) and two commercial juices (orange–clementine and bergamot–clementine). A cola soft drink and a bergamot juice, depleted of flavonoids, were employed as blank matrices to create fortified samples and thereby assess the accuracy of the methodology. Both juices and fruits were purchased in local shops and stored at 4 °C prior to instrumental analysis.

### 2.3. Sample Preparation

Prior to PS-MS/MS analysis, each juice sample was centrifuged at 12,000 rpm for three minutes. The supernatant was diluted 10-fold in water, and the internal standard, quercetin-3-*O*-glycoside, was added at a concentration of 5 mg/L. For HPLC-MS/MS determination, the samples were diluted 500-fold, and caffeic acid was added as an internal standard at a final concentration of 100 μg/L [30].

### 2.4. PS-MS/MS Analysis

The MS/MS experiments were carried out with a TSQ Quantum Vantage (Thermo Fisher Scientific, San José, CA, USA) triple-stage quadrupole mass spectrometer equipped with a homemade paper spray ionization source. For all determinations, qualitative Whatman filter paper n°1 (pore size 11 μm, thickness 180 μm) was used. The sample spotting volume was 15 μL, and once dried, the paper triangle was wetted with the same volume of methanol to allow the desorption of the ions. The PS-MS/MS working conditions were the following: negative ionization mode using a 5.0 kV voltage, with vaporizer and capillary temperatures at, respectively, 280 °C and 290 °C. The collision gas applied was argon at a pressure in the collision cell (Q2) of 1.5 mTorr, and the mass resolution at the first (Q1) and third (Q3) quadrupoles was set a 0.7 Da at full width at half-maximum. The scan time was set at 0.4 s and the number of micro scans at 2. The collision energy (CE) was optimized per compound and set at 25 eV for all three couples of isomers, while the Slens values were set at 160 V for each analyte. The analyses were performed by using the multiple reaction monitoring (MRM) scan mode, following specific gas phase transitions from the deprotonated precursor ion [M-H]^−^ as shown in Table 1. The acquisition time for each experiment was 2 min.

### 2.5. HPLC-MS/MS Analysis

The HPLC-MS/MS analyses were performed using a Thermo Scientific UHPLC instrument coupled to a TSQ Quantum Vantage (Thermo Fisher Scientific, San José, CA, USA) triple-stage quadrupole mass spectrometer. The chromatographic separation was achieved employing a C_18_ reversed-phase column (Hypersil GOLD, 2.1 × 100 mm, 3 μm, Thermo Fisher Scientific, San José, CA, USA). The sample injection volume was 5 μL, while the flow rate was set at 0.3 mL/min, using as elution solvents 0.1% HCOOH in water (solvent A) and methanol (solvent B) under gradient conditions. The gradients steps were the following: 5% B in isocratic for 1.5 min, from 5% to 30% B (1.5–3.5 min), 30% B isocratic for 1 min, from 30% to 40% B (4.5–8 min), 40% B isocratic for 1 min, from 40% to 60% B (9–11 min), 60% B isocratic for 1 min, from 60% to 70% B (12–14 min), 70% B isocratic for 1 min, from 70% to 80% B (15–16 min), 80% B isocratic for 2 min, from 80% to 5% B (18–20 min), and then an isocratic flow for 6 min to equilibrate the system before starting a new analysis. The total run time was 26 min. The MS/MS analysis was performed on a triple quadrupole mass analyzer with an ESI source operating in negative mode. The instrument conditions were the following: spray voltage = 4 kV, vaporizer and capillary temperature = 270 °C, and sheath and auxiliary gas = 40. For the MS/MS experiment, the parameters were the same as reported in Section 2.4 for the PS-MS/MS analyses.

### 2.6. Matrix Effect

The matrix effect was calculated using the following Equation (1):

ME (%) = (*B*/*A*) × 100 (1)

where *A* is the signal area of the analyte obtained from the analysis of a standard solution, and *B* the analyte signal in a blank matrix [31].

### 2.7. Data Analysis

The results obtained from PS-MS/MS and HPLC-MS/MS analyses were submitted to statistical analysis using Microsoft Excel’s Data Analysis according to Student’s *t*-test (α = 0.05). The test was performed to verify the ability of the methodology to discriminate in the quantification of flavanone-*O*-glycoside isomers and the effective comparability between the data obtained from the two different instrumental analyses.

### 2.8. Antioxidant Activity

The antioxidant capacity of the flavonoids under investigation was assessed using the DPPH radical scavenging assay, as already reported in our previous study [31]. The antixidant activity was evaluated spectrophotometrically by measuring the reduction in DPPH absorbance at 517 nm, after its reaction with the antioxidant molecules. The DPPH solution was prepared in methanol at 0.1 mM, while each flavonoid solution was in a concentration range from 2 to 140 µmol/L.

The experiments were conducted as follows: 100 µL of each standard solution was mixed with 100 µL of methanol and 1.8 mL of the 0.1 mM DPPH radical methanolic solution. The mixtures were shaken, kept in the dark at room temperature, and the DPPH absorbance was measured in triplicate after 120 min. The results were expressed as the percentage inhibition of DPPH radical by the calculation (2):

I% = [(A_0_ − A)/A_0_] × 100 (2)

where A_0_ is the absorbance value in the absence of an antioxidant (blank), and A is the absorbance value in the presence of an antioxidant compound. Spectrophotometric analysis was carried out using a UV/Vis Spectrophotometer Cary 50 Scan (Varian Inc., Palo Alto, CA, USA).

## 3. Results

In our previous study dealing with the assay of phenols by paper spray mass spectrometry [32], the content of flavanone *O*-glycosides in citrus juices was given by the sum of the concentration of the two positional isomers, i.e., the 7-*O*-neoesperidosides and the 7-*O*-rutinosides; in fact since there were no chromatographic separation, it was not possible to differentiate between the two isomeric forms. Starting from this study, the present work aimed to develop a different, rapid, and accurate method, still based on paper spray mass spectrometry, but capable of distinguishing the two isomers and determining their ratio in citrus beverages.

### 3.1. Study of MS/MS Spectra for Isomers

**Figure 1:**
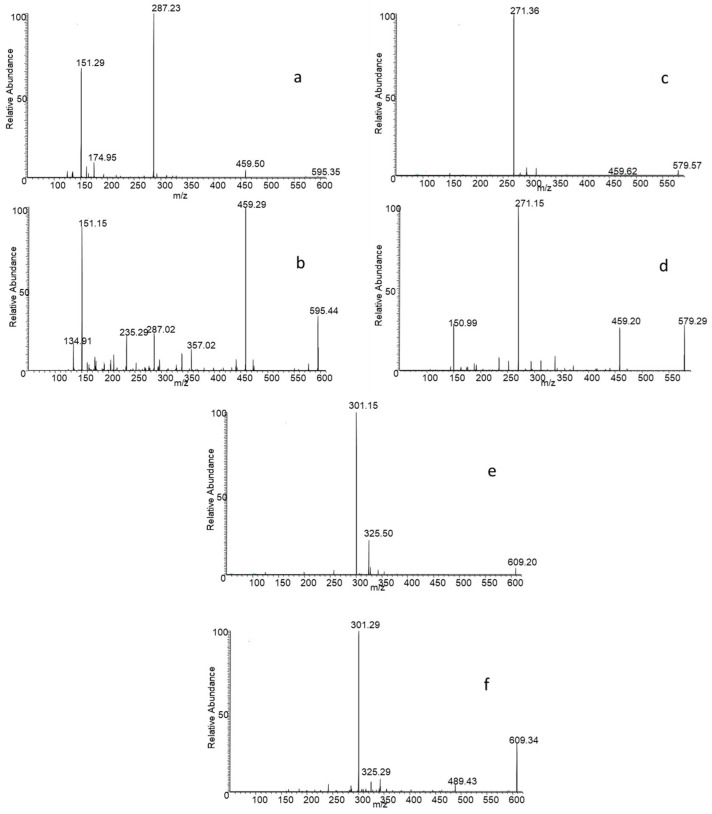
MS/MS spectra of eriocitrin (**a**), neoeriocitrin (**b**), narirutin (**c**), naringin (**d**), hesperidin (**e**), neohesperidin (**f**).

The new protocol relies on the distinct fragmentation patterns exhibited by the flavonoid isomers under investigation, as demonstrated by an in-depth analysis of their CID-MS/MS spectra. In particular, it was observed that the ratio of the intensity of the ion signals coming from the product ions provided by the two isobaric species varies hanging on the structures of the isomer. As already observed in our previous work, this variation is noticeable for the isomer pair of eriocitrin (eriodictyol-7-*O*-rutinoside) and neoeriocitrin (eriodictyol-7-*O*-neohesperidoside) [32]. In this case, the MS/MS spectra, acquired in negative mode using a collision energy of 25 eV, exhibit two distinct base peaks: neoeriocitrin fragmentation provides the base peak at *m*/*z* 459, which probably arises from the aglycone C-ring break, while the fragmentation of eriocitrin shows a base peak at *m*/*z* 287, corresponding to the formation of the deprotonated eriodyctiol (see Figure 1).

The difference in the fragmentation profile for the other two pairs of isomers at 25 eV collision energy is not so marked; in fact, the base peak is in all cases given by the formation of the deprotonated aglycone, i.e., the product ion at *m*/*z* 271 for the naringenin diglycosides, and the ion at *m*/*z* 301 for the hesperetin diglycosides. For both pairs, the difference lies in the relative intensity (RI) of the other product ions. For instance, in the case of naringin-narirutin isomer pair, the RI of the product ion at *m*/*z* 459 decreases significantly in the spectrum of rutinoside. The reason is that, in the case of naringin, the ion at *m*/*z* 459 can be generated by two possible fragmentations; the first one involves the C-ring breakage, and the second one the fragmentation of ring glucose moiety. Conversely, deprotonated narirutin only fragments generating the aglycone ion.

For the pair hesperidin–neohesperidin, the CID-MS/MS spectra show a common fragment at *m*/*z* 325, whose RI increases for the rutinoside isomer. In contrast, the ion signal at *m*/*z* 489, which corresponds to an internal fragmentation of glucose, appears exclusively in the neohesperidoside spectrum. Figure S1 shows the possible fragmentation of the neohesperidoside isomers.

We chose to use 25 eV as optimal collision energy, because at higher energies an excessive fragmentation of the rutinoside isomers was observed, limiting the sensitivity of the analysis.

### 3.2. Method Validation

The assay of the total concentration of flavonoid pairs was conducted under the MRM condition, monitoring the same gas-phase transitions previously reported [24], while the evaluation of the percentage ratio between the isomers needed more than one transition channel (Table 1).

**Figure 2:**
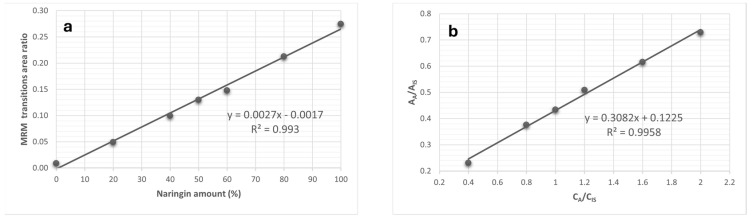
Calibration curves used to discriminate the isomers naringin and narirutin (**a**), and to quantify their total amount (**b**)—The MRM transitions area ratio is referred to in Table 1, while A_a_ and A_is_ are, respectively, the areas due to the current of the MRM transitions of the analyte and the internal standard, and C_a_ and C_is_ are their concentrations.

For example, to assess the percentage of eridictiol-7-*O*-glycoside isomers (neoeriocitrin and eriocitrin), the ratio between the area of the transition *m*/*z* 595 → *m*/*z* 459 and the sum of the areas of all observed transitions, was considered. In the case of the naringin/narirutin pair, the area of the transition *m*/*z* 579 → *m*/*z* 459 was evaluated against the sum of all transition areas (Figure 2). Lastly, for neohesperidin and hesperidin, the ratio of the transition area *m*/*z* 609 → *m*/*z* 325 to the total area of all followed transitions was taken into account (see Table 1).

For each isomer pair, the ratio calibration curves were built by preparing eight solutions with increasing total concentration of both species, but a variable ratio between rutinosides and neohesperidosides. In particular, the first solution prepared at 10 mg/L, was composed only of neohesperidosides (0–100% ratio), the second at 8 mg/L contained 20% rutinosides and 80% neohesperidosides (20–80%), while the other standard solutions were made at 6 mg/L using 40–60% ratio, 5 mg/L using a 50–50% ratio, 4 mg/L using a 60–40% ratio, 2 mg/L using a 80–20% ratio; finally, a solution at 10 mg/L composed solely of rutinosides (100–0% ratio). Using the set of data coming from the analyses of the above-mentioned standard solutions, it was possible to build two different calibration curves for each isomer pairs: the first one, by plotting the ratio of the MRM transition of each isomer vs. the % ratio, and the second obtained by reporting the intensity of each MRM analyte vs. the concentration. An internal standard (Quercetin-3-*O*-glucoside) at 5 mg/L concentration was added to all standard solutions; by this approach, it was possible to obtain the total flavonoid concentration as the sum of the two isomers present in each pair, following the method previously reported [32], and, in parallel, to determine the ratio between the two isomers.

For all isomer pairs under study, the method demonstrated a good linearity in the instrumental response; in fact, each calibration curve displayed a linear correlation coefficient exceeding 0.98. Figure 2 represents the calibration curves retrieved for the naringin/narirutin pair.

After the evaluation of linearity, the reproducibility of the calibration curves was assessed by preparing and analyzing two solutions at two representative ratios (80–20% and 20–80% neohesperidosides–rutinosides) three times over the period of one week. In every instance, the analyses yielded an instrumental response in accordance with the levels of calibration curves, showing RSD% values less than 15% indicating a high degree of reproducibility.

The matrix effect was assessed by analyzing a blank matrix suitably diluted at different levels, and enriched with 10 mg/L of diglycosides composed by 80% neohesperidosides and 20% rutinosides. In particular, the matrix used was cola diluted 20 times (S1), 10 times (S2), 5 times (S3), and twice (S4). The obtained values are shown in Table 2.

For the neoeriocitrin–eriocitrin isomer pair, the calculated accuracy values were all near to 100% showing no matrix effect at any dilution level. A similar trend was noted for the neohesperidin–hesperidin pair, where the accuracy remained acceptable even for the spiked samples prepared with a two-fold diluted matrix (80%), whereas, in the case of the naringin–narirutin isomer pair, the accuracy decreased significantly, reaching 70% for the samples prepared with the more concentrated matrix denoting a significant matrix effect. The matrix effect was accurately calculated by analyzing all the fortified samples five times, and comparing the instrumental responses with those obtained from the analysis of standard solutions at the same concentration and analytes ratio (Table 2). The ratio of the responses close to 1 means that the influence of the matrix on the analysis can be considered negligible. Table 2 also shows that the calculated matrix effect is effectively stronger for sample S3 and S4 of the pair naringin and narirutin. Given these results, and due to the possibility that in real cases some pairs may be present at very low concentrations, it was decided to proceed by accomplishing the calibration curves in matrix, using standard solutions prepared in cola diluted 10 times. Again, the linearity was quite good for all flavonoid pairs.

The performance of the quantification process was then evaluated by analyzing a further set of six fortified samples to determine the accuracy and reproducibility of the analytical procedure. As the calibration curves were already made using the cola-based beverage, a different matrix was chosen for the analysis of the accuracy parameter. Specifically, bergamot juice was filtered through a C_18_ cartridge to trap all the flavonoids, and the water-eluted fraction was subsequently used as blank matrix. The accuracy was evaluated by analyzing six fortified blank samples, prepared as a percentage ratio at the outmost of the standard curves used to retrieve the ratio of isomers. In detail, each sample was prepared at a total concentration of 10 mg/L, with different ratios of neohesperidosides to rutinosides isomers, as specified below: 100:0 (FS1), 90:10 (FS2), 10:90 (FS3), 0:100 (FS4), and two intermediate samples at 95:5 (FS5) and 5:95 (FS6). The results are reported in Table 3. Almost all accuracy values are acceptable except for FS5 and FS6 that show accuracy values higher than 120%.

Furthermore, in order to show the ability of the presented protocol to discern between very similar isomer percentages, the difference in instrumental response was examined using the t-test (α = 0.05) on the results of the analysis from the same fortified samples. The test revealed *p* values ≤ 0.05 for neoeriocitrin and eriocitrin in all the samples examined, suggesting the existence of significant differences among all the instrumental measurements. Conversely, for the pairs neohesperidin–hesperidin and naringin–narirutin, the t-tests produced a *p* value ≥ 0.05 when the difference between the percentage was only 5%. This means that the methodology does not allow the differentiation between samples, which present a difference in less than 10% in the content of pairs neohesperidin–hesperidin, and naringin–narirutin.

The table also includes reproducibility values, which were determined by performing tests on two fortified samples three times over a period of one week. In both cases, the RSD% is less than 13%, indicating a high level of reproducibility in the measurements.

### 3.3. Method Application to Real Samples

After validation of the analytical method, the developed protocol was applied to seven real samples of citrus juices and the results are shown in Table 4. The same samples were submitted to HPLC-MS/MS analysis to verify the data obtained and thus test the robustness and reliability of the methodology (Figure S2). A t-test (α = 0) was performed between the PS-MS/MS and the HPLC-MS/MS results for the clementine–bergamot juice that contains all the six flavanone-O-glucosides analyzed. The test gave *p* value ≥ 0.05 for all the analytes, indicating the absence of significant difference between the data.

## 4. Discussion

The present study introduces an effective advancement in the analysis of citrus juices and beverages by developing a rapid and accurate method to assay the single isomeric forms of *O*-glycoside flavanones. The use of paper spray mass spectrometry offers many advantages, including reduced sample preparation and increased throughput over traditional chromatographic techniques. As a matter of fact, the length of the acquisition experiment is 2 min, which must be added to a very fast step of sample preparation which involves rapid centrifugation and a suitable dilution. It is worth noting that there is also an efficient and sustainable use of solvent whose amount is limited to a few microliters of MeOH needed to achieve the ionization. By this methodology, it is possible to retrieve, using the same acquisition data, the concentration of valuable antioxidants such as diglycosides flavanones in citrus, which exist in two isomeric forms and also the amount of each single isomer, which differ for their glycoside moieties, i.e., neohesperidose and rutinose.

Furthermore, this study highlights the importance of understanding the fragmentation patterns of the target analytes, which are crucial for selecting appropriate MRM transitions and ensuring accurate quantification. In fact, the fragmentation patterns observed in CID-MS/MS spectra give a mechanistic basis for discrimination of the positional isomers, highlighting the sensitivity of the technique to slight structural differences.

The developed approach was successfully validated by calculating all analytical parameters, demonstrating good linearity, reproducibility, and accuracy, as evidenced by the high correlation coefficients, low RSD% values, and an acceptable accuracy. The observed matrix effects emphasize the importance of evaluating matrix composition when developing analytical methods for complex samples, such as citrus juices, which is fundamental when mass spectrometry is used in conjunction with non-isotopic internal standards. The construction of the ratio calibration curve in the specific matrix successfully reduced the impact of matrix effects on the results’ accuracy.

Furthermore, the application of the method to real samples corroborates its practical utility, and the comparison with HPLC-MS/MS results provides further confidence in the accuracy and reliability of the PS-MS/MS analytical approach. This approach may be useful also in discriminating the ingredients of a commercially prepared juice, which usually possess a well definite presence of rutinose and/or neohesperidose derivatives. For example, in the case of bergamot juice, it would allow the testing of potential adulteration with lemon juice by accurately quantifying the proportions of neohesperidine and hesperidin. Because bergamot contains only neohesperidine, while lemon is composed mainly of hesperidin, any variation in the ratio of these two molecules indicates the presence of other fruits in the product.

In order to investigate the nutraceutical action of the single positional isomers and their possible role in adding value to a specific juice, we decided to evaluate the antioxidant activity of the pure standard by the conventional chemical methodology based on the DPPH assay. As previously demonstrated in our earlier study, the antioxidant activity of neohesperidoside flavonanones was found to be strongly concentration-dependent and closely related to their chemical structure. In particular, the ability to quench the DPPH radical was influenced by the nature of the substituents on the aglycone portion of the molecule [22]. Those data have been now updated by the results obtained for rutinoside analogs. Again, the glycosides of eriodictyol exhibited the highest antioxidant activity, followed by those of hesperetin, and finally by naringenin conjugates. In particular, the antioxidant activity of flavanone isomers was evaluated in depth at three concentrations (4 µM, 8 µM, and 18 µM). As shown in Table 5, neohesperidosides exhibited significantly superior antioxidant activity compared to the corresponding rutinosides at lower concentrations. This difference decreases at increasing concentrations, disappearing at 40 µM, where a plateau in antioxidant activity is observed. The latter findings, together with the ability to quickly and accurately analyze the different antioxidant species, make this methodology an innovative and promising approach for assessing the quality of citrus juices.

## 5. Conclusions

This manuscript introduces an innovative analytical approach for direct isomer ratio determination of flavanone glycosides, specifically rutinosides and neohesperidosides, which are key antioxidant compounds of citrus juices. The isomers under study include neoeriocitrin and eriocitrin, naringin and narirutin, neohesperidin and hesperidin. The proposed methodology is based on the paper spray tandem mass spectrometry, which provides a very rapid, yet reliable, determination. Its reliability and specificity are further enhanced by the use of the MRM acquisition mode. Moreover, the DPPH assay, which is a common method for evaluating radical scavenging capability, demonstrated a higher antioxidant activity by the neohesperidose derivatives when compared to that of rutinose analogs. This innovative analytical approach significantly improves the efficiency of determining flavonoid isomers in citrus juices and related products. It offers a rapid, reliable, and sensitive method for quantifying these important compounds, contributing to the quality control of citrus-based products.

## Figures and Tables

**Table 1:** MRM transitions monitored.

Analyte	Transition	CE (eV)
Neoeriocitrin/Eriocitrin	*m*/*z* 595 ˃ *m*/*z* 287 *m*/*z* 595 ˃ *m*/*z* 459	25
Naringin/Narirutin	*m*/*z* 579 ˃ *m*/*z* 271 *m*/*z* 579 ˃ *m*/*z* 459	25
Neohesperidin/Hesperidin	*m*/*z* 609 ˃ *m*/*z* 301 *m*/*z* 609 ˃ *m*/*z* 325 *m*/*z* 609 ˃ *m*/*z* 489	25
Quercetin-3-*O*-glucoside (IS)	*m*/*z* 463 ˃ *m*/*z* 301	22

**Table 2:** Values of accuracy and matrix effect.

Spiked Samples 80% Neohesperidosides 20% Rutinosides	S1	S2	S3	S4
Neoeriocitrin Found (%)	78.6 ± 0.1	75.6 ± 0.5	80.5 ± 0.6	74.2 ± 1.1
Accuracy (%)	96	95	101	93
Matrix effect	0.99	1.02	0.99	0.98
Naringin Found (%)	77.3 ± 1.2	68.4 ± 1.3	57.8 ± 0.6	56.1 ± 0.6
Accuracy (%)	97	86	72	70
Matrix effect	0.94	0.81	0.63	0.60
Neohesperidin Found (%)	72.1 ± 7.5	72.1 ± 1.9	71.0 ± 3.2	65.8 ± 7.7
Accuracy (%)	90	90	89	82
Matrix effect	0.92	0.92	0.91	0.85

**Table 3:** Values of accuracy and reproducibility.

	Neoeriocitrin/ Eriocitrin	Naringin/ Narirutin	Neohesperidin/ Hesperidin		RSD% *
Fortified Samples (Neohesperidosides: Rutinosides)	Accuracy (%)	Accuracy (%)	Accuracy (%)		
FS1 (100:0)	101/100	103/100	102/100	FS2 (90:10)	8.2
FS2 (90:10)	102/91	91/92	100/102	FS3 (10:90)	6.3
FS3 (10:90)	103/90	106/90	100/100		
FS4 (0:100)	102/98	95/105	118/98		
FS5 (95:5)	100/96	91/˃120	99/115		
FS6 (5:95)	106/95	˃120/95	˃120/93		

* The reproducibility was determined by analyzing two samples (FS2 and FS3) three times over a period of one week.

**Table 4:** Flavanones glycosides amount (mg/L) found in real samples and the ratio between neohesperidosides and rutinosides.

	Neoeriocitrin/ Eriocitrin		Naringin/ Narirutin		Neohesperidin/ Hesperidin	
	Concentration (mg/L)	Ratio (%)	Concentration (mg/L)	Ratio (%)	Concentration (mg/L)	Ratio (%)
Citron	15 ± 1	0:100	-	-	9 ± 1	0:100
Grapefruit	-	-	319 ± 34	75:25	-	-
Bergamot	76 ± 10	100:0	72 ± 8	100:0	49 ± 5	100:0
Orange	-	-	27 ± 3	0:100	86 ± 8	0:100
Lemon	106 ± 4	5:95	14 ± 1	30:70	185 ± 5	10:90
Orange–Clementine	-	-	37 ± 3	10:90	21 ± 2	100:0
Clementine–Bergamot	80 ± 6	85:15	75 ± 6	84:16	134 ± 7	52:48

**Table 5:** DPPH inhibition values (%).

Concentration (µM)	DPPH Inhibition (%)					
	Neoeriocitrin	Eriocitrin	Naringin	Narirutin	Neohesperidin	Hesperidin
4	22	19	3	0.3	6	2
8	48	37	4	0.5	13	3
18	100	70	5	4	40	37

## Data Availability

The original contributions presented in this study are included in the article and Supplementary Materials. Further inquiries can be directed to the corresponding author.

## Supplementary Materials

The following supporting information can be downloaded at: https://www.mdpi.com/article/10.3390/antiox14010020/s1.
