# Supplementary material for: Direct Determination of Flavanone Isomers in Citrus Juice by Paper Spray Tandem Mass Spectrometry
Source: Antioxidants (Basel). 2024 Dec 27;14(1):20. doi: 10.3390/antiox14010020 (PMC11761641; doi:10.3390/antiox14010020)
Supplement: Supplementary file 1 [file antioxidants-14-00020-s001.zip › antioxidants-3346874-supplementary.pdf]

Direct Determination of Flavanone Isomers in Citrus Juice by Paper Spray Tandem Mass Spectrometry

Supplementary material

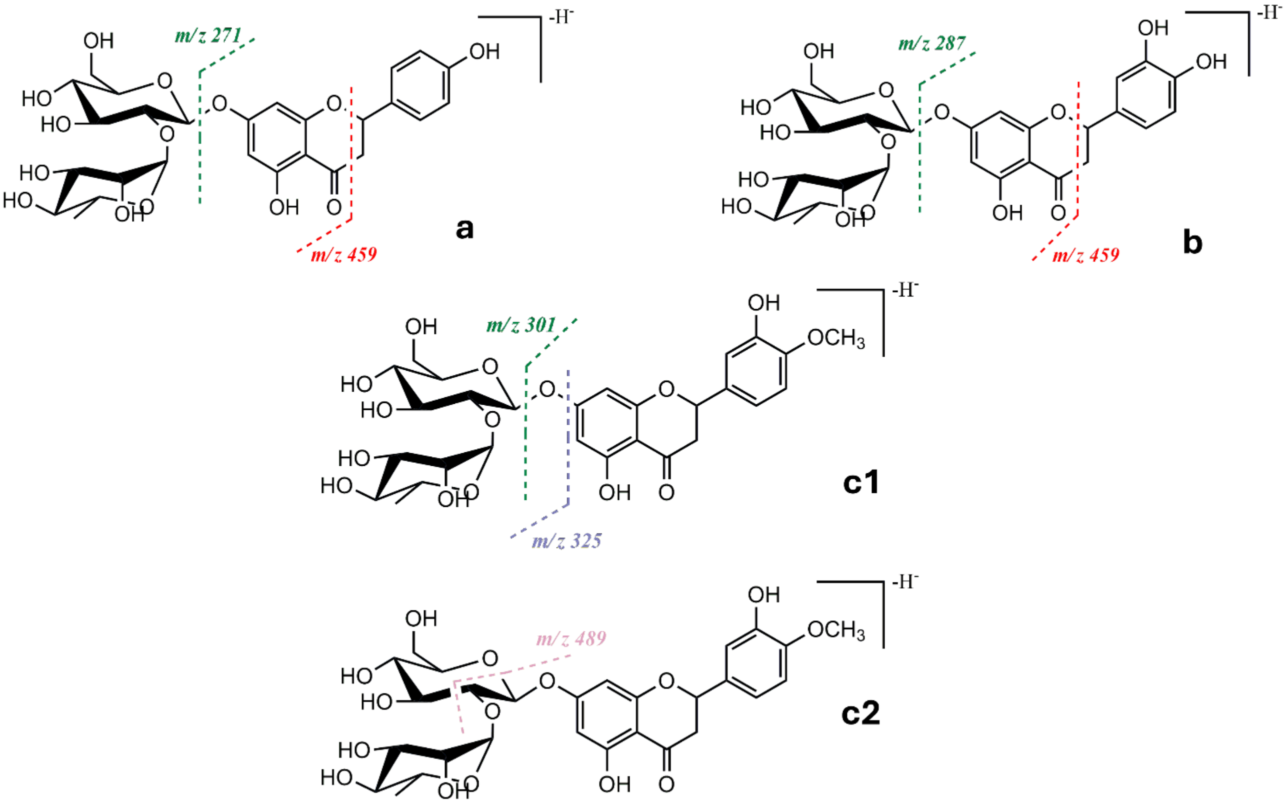

Figure S1 Proposed fragmentation for a) naringin, b) neoeriocitrin, c) neohesperidin.

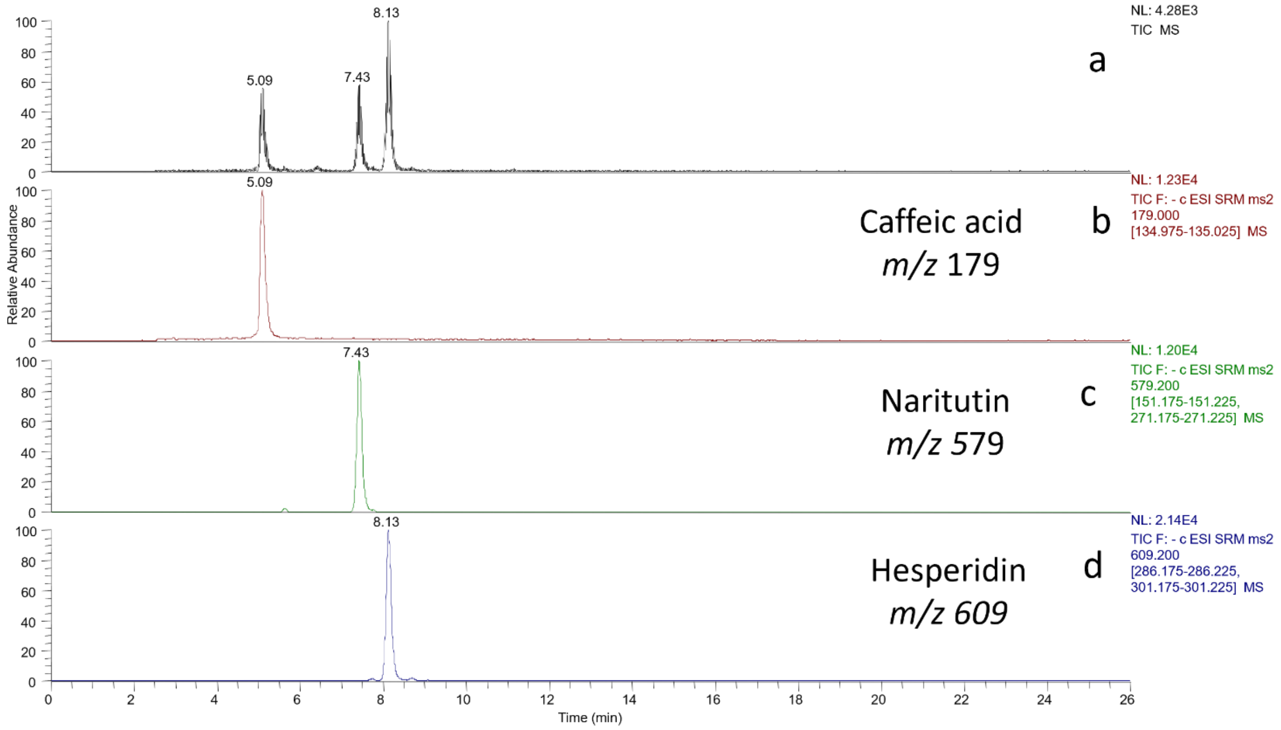

Figure S2 Representative HPLC- MS/MS chromatogram of the orange juice sample: a) TIC MS, b) EIC of caffeic acid, c) EIC of narirutin; d) EIC of hesperidin.
